# Preoperative multiparameter MRI‐based prediction of Ki‐67 expression in primary central nervous system lymphoma

**DOI:** 10.1002/pro6.70005

**Published:** 2025-03-18

**Authors:** Jian Xu, Lili Zhang, Qingzeng Liu, Jian Zhu

**Affiliations:** ^1^ Shandong Cancer Hospital and Institute Shandong First Medical University and Shandong Academy of Medical Sciences Jinan P. R. China; ^2^ Shandong Provincial Key Medical and Health Laboratory of Pediatric Cancer Precision Radiotherapy (Shandong Cancer Hospital) Jinan P. R. China; ^3^ Liaocheng People's Hospital of Shandong Province Liaocheng P. R. China

**Keywords:** Ki‐67, Lymphoma, Machine Learning, Magnetic Resonance Imaging, Radiomics

## Abstract

**Background:**

Ki‐67 is a key marker of tumor proliferation. This study aimed to develop machine learning models using single‐ and multi‐parameter MRI radiomic features for the preoperative prediction of Ki‐67 expression in primary central nervous system lymphoma (PCNSL), aiding prognosis and individualized treatment planning.

**Methods:**

A retrospective analysis of 74 patients was conducted using MRI scans, including T1, contrast‐enhanced T1, T2, T2‐FLAIR, DWI, and ADC sequences. Patients were categorized into high‐expression (Ki‐67 > 70%) and low‐expression (Ki‐67 ≤ 70%) groups. Tumor volumes of interest (VOIs) were manually delineated by radiologists, and 851 radiomic features were extracted using 3DSlicer. After preprocessing, including bias field correction and normalization, feature selection was performed using SelectKBest and ANOVA. Eight machine learning classifiers, including Logistic Regression, Random Forest, and SVM, were applied to single‐ and multi‐parameter datasets.

**Results:**

Multiparameter models, particularly Naive Bayes and Logistic Regression, demonstrated superior predictive performance (AUC: 0.78, 0.73; AP: 0.90, 0.83) compared to single‐parameter models. Decision curve analysis highlighted that Logistic Regression provides the highest net benefit, followed by Naive Bayes.

**Conclusion:**

Multiparameter MRI models are more accurate and stable for predicting Ki‐67 expression in PCNSL, supporting clinical decision‐making.

## Graphical abstract

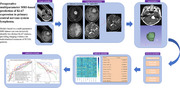

## 1 INTRODUCTION

Primary central nervous system lymphoma (PCNSL) is an extranodal non‐Hodgkin lymphoma (NHL) affecting the brain, spinal cord, cerebrospinal fluid, or vitreoretinal compartments, with the vast majority of cases classified as diffuse large B‐cell lymphoma (DLBCL). PCNSL is a rare tumor with an annual incidence rate of approximately 0.4 per 100, 000 people, which increases with age to as high as 4 per 100, 000 in individuals over 70.^1^ PCNSL accounts for 4%–6% of all extranodal lymphomas and 4% of newly diagnosed malignant brain tumors.^2^ Although PCNSL can occur at any age, the median age of onset is 56–61 years. For patients aged 19 years, the 1‐year, 5‐year, and 10‐year survival rates are 83%–88%, 74%–78%, and 66%–73%, respectively. For those aged 20–64 years, the corresponding survival rates are 40%–60%, 29%–37%, and 23%–27%. In elderly patients (aged 65 years or older), the prognosis is worse, with estimated 1‐year, 5‐year, and 10‐year survival rates of 33%–48%, 13%–24%, and 10%–13%, respectively.^3^ For immunocompetent adults, the median overall survival (OS) is approximately 25 months.^4^ In cases of immunosuppression, such as patients with HIV/AIDS or transplantation, the risk of developing Epstein–Barr virus (EBV)‐associated PCNSL increases.^5^

Ki‐67 is a nuclear antigen expressed during the cell proliferation phase that accurately reflects cellular proliferative activity.^6^, ^7^ It has significant prognostic value in predicting tumor classification and outcomes.^8^, ^9^, ^10^ A study by Li et al. indicated that patients with DLBCL with the nongerminal center B‐cell‐like subtype (GCB) and high Ki‐67 expression had 3‐year OS and progression‐free survival rates of only 65.2% and 56.4%, respectively, which were significantly lower than those of patients with low Ki‐67 expression.^11^ Han et al. developed a specific prognostic model that further validated the significance of Ki‐67 in the OS for patients with DLBCL. Based on data from 1, 070 patients with DLBCL, the model demonstrated that Ki‐67 expression level can serve as an independent prognostic factor in multivariate analysis, particularly in patients receiving R‐CHOP or similar regimens, providing better predictive efficacy for high‐risk patients than the traditional International Prognostic Index (IPI).^12^

Ki‐67 expression can only be determined by the immunohistochemistry of biopsies or surgically obtained pathological specimens. However, a biopsy is an invasive procedure and may lead to complications, such as bleeding and needle tract seeding. Additionally, heterogeneity within the puncture sample and sampling limitations may prevent the Ki‐67 index from fully representing the entire lesion, thereby limiting analytical accuracy. Moreover, immunohistochemistry is time‐consuming and does not allow real‐time assessment of the Ki‐67 index. Therefore, finding a noninvasive, more comprehensive, and accurate method for predicting Ki‐67 expression in patients with PCNSL is essential.

Radiomics has recently been used to analyze and predict Ki‐67 expression in various malignant tumors, demonstrating good predictive performance. Bi et al. successfully used multiparameter MRI radiomic features to predict the Ki‐67 proliferation status of nasopharyngeal malignancies, achieving an AUC of 0.852 and accuracy of 86.3% on the test dataset. These results suggest that multiparameter MRI radiomics may be a noninvasive and reliable tool for predicting Ki‐67 proliferation status in nasopharyngeal malignancies.^13^

Wu et al. developed a CT‐based radiomics nomogram for predicting Ki‐67 expression in patients with hepatocellular carcinoma (HCC), incorporating AFP levels and Edmondson grade. Radiomic features were significantly associated with Ki‐67 expression, and the nomogram showed AUCs of 0.884 and 0.819 in the training and validation groups, respectively, demonstrating good predictive ability and providing guidance for personalized treatment and clinical monitoring of HCC patients.^14^ Zhao et al. applied machine learning techniques to predict the Ki‐67 proliferation index in meningioma patients based on MRI radiomic features. This study included 371 meningioma patients and used various radiomic features to build a prediction model, achieving AUCs of 0.837 and 0.700 on the internal and external validation sets, respectively, indicating high predictive accuracy.^15^ Li et al. analyzed the HER‐2 and Ki‐67 statuses of breast cancer patients using dynamic contrast‐enhanced MRI images. Their results showed that radiomic features in both the intratumoral and peritumoral areas could effectively predict Ki‐67 status, with AUCs on the validation set of 0.713 and 0.749, respectively, underscoring the potential of MRI radiomics for breast cancer prognosis.^16^

However, owing to the rarity of PCNSL, few radiomics studies have focused on this tumor type. Therefore, this study aimed to develop and validate an MRI‐based multiparametric radiomics model for preoperative evaluation of Ki‐67 proliferation status in PCNSL.

## 2 METHODS

### 2.1 Study design

**FIGURE 1:**
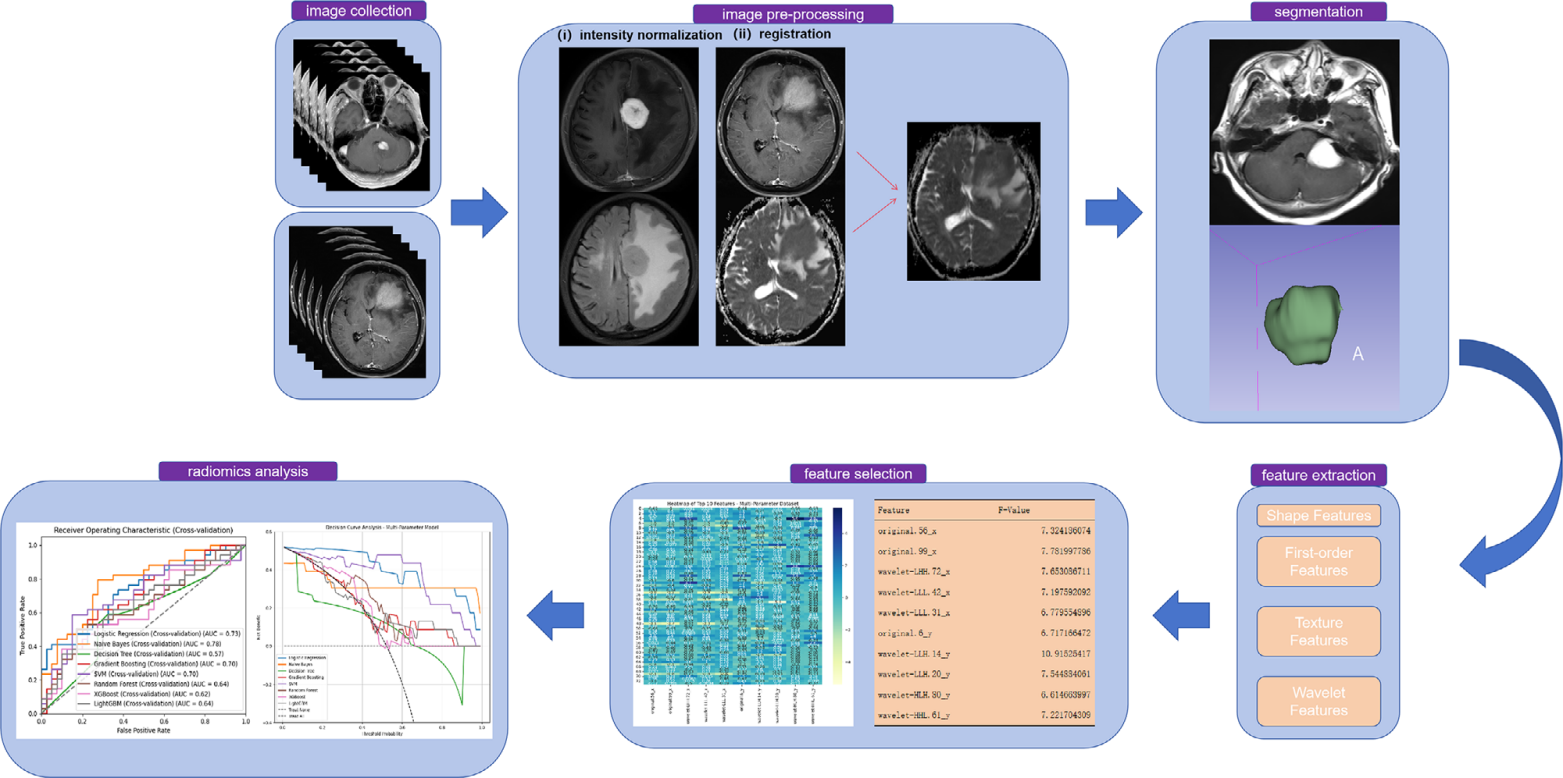
Research workflow. Images were collected, preprocessed, and annotated, followed by feature extraction, selection, and, finally, model building and evaluation.

Figure 1 provides an overview of the study workflow. The workflow for image processing and radiomics analysis is illustrated in Figure 1. The process begins with image collection, where MRI data from multiple sequences (e.g., T1 and T2) are collected. Preprocessing includes intensity normalization to reduce variability and image registration to align scans within a consistent spatial framework. Tumor segmentation is then performed, isolating regions of interest manually. Following segmentation, radiomic features are extracted, categorized into shape, first‐order, texture, and wavelet‐based features, capturing tumor geometry, intensity distribution, and textural patterns. A feature selection step uses univariate analysis of variance (ANOVA) to identify the most relevant features. Finally, radiomics analysis is conducted, employing machine learning models to evaluate feature significance and predict outcomes, with model performance visualized through receiver operating characteristic (ROC) curves. Decision curve analysis shows the net benefit of each classifier at different decision thresholds. This streamlined process ensures robust and reproducible data analysis.

### 2.2 Patient characteristics and MR examination

This study retrospectively collected data from 253 PCNSL patients treated at Shandong Cancer Hospital and Institute between January 2011 and June 2024.

The inclusion criteria were as follows: (1) histologically confirmed PCNSL (according to the 2021 WHO Classification of CNS Tumors)^17^ and (2) availability of pretreatment MRI images. The exclusion criteria were as follows: (1) absence of pathological and immunohistochemical results, (2) incomplete MRI sequences and/or significant motion artifacts in the scans, (3) unrelated history of intracranial disease, and (4) history of surgery or radiotherapy/chemotherapy prior to MRI.

Ultimately, the MRI data from 74 patients were retained, encompassing 1, 112 sequences and totaling 30,176 images. The selected imaging modalities included complete contrast‐enhanced T1, T1‐weighted imaging (T1WI), T2‐weighted imaging (T2WI), T2 fluid‐attenuated inversion recovery (T2_FLAIR), diffusion‐weighted imaging (DWI), and apparent diffusion coefficient (ADC) images, with a total of 8,724 images analyzed. All patients presented with PCNSL lesions within the brain parenchyma, with no systemic involvement, as confirmed by CT‐MRI scans of the chest, abdomen, and pelvis. Additionally, there was no evidence of immunodeficiency disorders or human immunodeficiency viral infection.

### 2.3 Image segmentation and feature extraction

Bias field correction was performed using SimpleITK to eliminate signal intensity variations caused by magnetic field inhomogeneities. The images were then resampled to a voxel size of 1 mm × 1 mm × 1 mm using B‐spline interpolation in scipy signal to reduce the impact of slice thickness variation and isotropic voxel effects. Subsequently, to minimize inherent pixel intensity differences across different MRI scanners, all image volumes were intensity‐scaled within a 0–255 range after removing outlier pixels.^18^

The preprocessed images were uploaded to the 3DSlicer platform (version 5.2.1), where two radiologists with over five years of experience manually annotated the volume of interest (VOI) of the tumor in the contrast‐enhanced T1 images. To assess the reproducibility of the radiomic features, 50 patients were randomly selected for a second segmentation by one of the radiologists after a one‐month interval. The two radiologists reached a consensus on VOI delineation, and patients for whom consensus could not be achieved were excluded.

After VOI delineation on the contrast‐enhanced T1 images, rigid registration was performed on the 3DSlicer platform using the General Registration (ANTs) module, with the contrast‐enhanced T1 images as fixed images and other sequences as moving images.

Following registration, feature extraction was performed on each sequence using the Radiomics module of the 3DSlicer platform. A total of 851 radiomic features were initially extracted from the VOI in each sequence, including shape, first‐order radiomic, and higher‐order radiomic features from four different matrices, including gray‐level cooccurrence matrix (GLCM), gray‐level run length matrix (GLRLM), and gray‐level size zone matrix (GLSZM). The GLCM was first demonstrated by Haralick and Shanmugam in the 1970s and is a recognized statistical tool for extracting texture information from images. The GLCM is used to analyze the spatial relationship between pixel intensities in an image. The GLRLM extracts higher‐order statistical texture features, as described by Galloway in 1975; it describes the length of consecutive pixels with the same gray level, helping to capture the structural features of the image.^19^ The GLSZM quantifies the size of zones with similar gray levels in an image, providing insight into texture patterns.

### 2.4 Feature preprocessing and selection

Each dataset was first checked for missing values, and mean imputation was set up to handle any overlooked missing data. Standardization of numerical features was performed using the StandardScaler module from the Scikit‐learn library in Python, scaling features to a standard normal distribution with a mean of 0 and standard deviation of 1.

**FIGURE 2:**
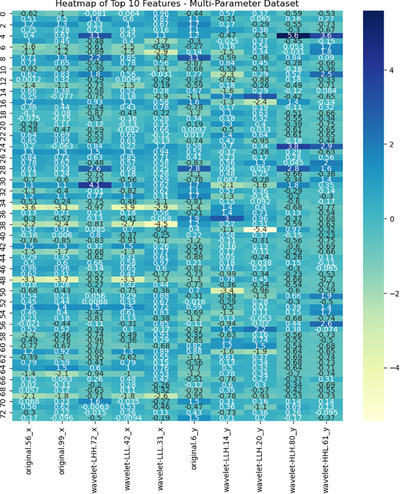
Heatmap of selected features: each column represents one of the 10 features, and each row represents a sample, totaling 74 samples. Colors indicate the magnitude of feature values. Dark to light colors represent negative, zero, and positive values, respectively, to display variations in feature values.

Feature selection was conducted using the SelectKBest module from the Scikit‐learn library in Python, selecting the top features most strongly correlated with the labels by applying univariate ANOVA (f_classif). This selection of the most relevant features enhances the sensitivity and accuracy of the model.^20^ A heatmap of the selected features from the multiparameter dataset is shown in Figure 2, and all selected features, along with their F values for the single‐ and multi‐parameter datasets, are presented in Appendix 1.

### 2.5 Model training and evaluation

The processed dataset was split into training and testing sets in a 7:3 ratio. Eight classical and modern machine learning algorithms were selected: Logistic Regression, Naive Bayes, Decision Tree, Gradient Boosting, Support Vector Machine, Random Forest, XGBoost, and LightGBM. Initially, the features retained from each sequence (T1, contrast‐enhanced T1, T2, T2‐FLAIR, DWI, and ADC) were used to build single‐parameter datasets, and each dataset was modeled using the eight classifiers. Next, using Python's pandas library, the above imaging feature files were merged according to patient ID, classification labels, and feature name fields to ensure data consistency and matching. The resulting multiparameter dataset was then subjected to the same feature selection and modeling methods with each of the eight classifiers.

After model building, the model performance was evaluated using the area under the receiver operating characteristic curve (AUC) and the area under the precision–recall curve (average precision; AP). Cross‐validation was conducted to obtain more stable evaluation results, and the classifier with the highest mean AUC was selected as the optimal classifier.^21^, ^22^, ^23^ Classifier performance within the multiparameter model was compared from different perspectives using DeLong's test and a confusion matrix. Finally, decision curve analysis was conducted to evaluate the net benefit of each classifier at different decision thresholds within the multi‐parameter model. The details are provided in Appendix 2.

## 3 RESULTS

### 3.1 Patient characteristics

This study included 74 patients (39 male, 35 female), with an average age of 63.49 years (63.49 ± 9.30 years). Previous studies have used a Ki‐67 expression index of 70% as the cutoff for PCNSL.^24^, ^25^ In this study, the median Ki‐67 index of the samples was 70.5%; therefore, the high‐Ki‐67 group was defined as patients with a Ki‐67 index > 70%, and the low‐Ki‐67 group was defined as patients with a Ki‐67 index≤70%. There were no statistically significant differences between the two groups in terms of age or sex (*p* > 0.05). The baseline clinical and MRI characteristics of the two groups are summarized in Table 1.

**TABLE 1:** High Ki‐67 index (n = 34)/Low Ki‐67 index (n = 40).

	High Ki‐67 index	Low Ki‐67 index
Gender (M/F)	13/21	26/14
Subtentorial involvement(Y/N)	8/26	8/32
Deep brain involvement(Y/N)	15/19	9/31
Number of lesions (Single/Multiple)	18/16	12/28

High Ki‐67 index, Ki‐67 >70%; Low Ki‐67 index, Ki‐67≤70%.

### 3.2 Performance of radiomic models

#### 3.2.1 Comparison between single‐ and multi‐parameter models

**FIGURE 3:**
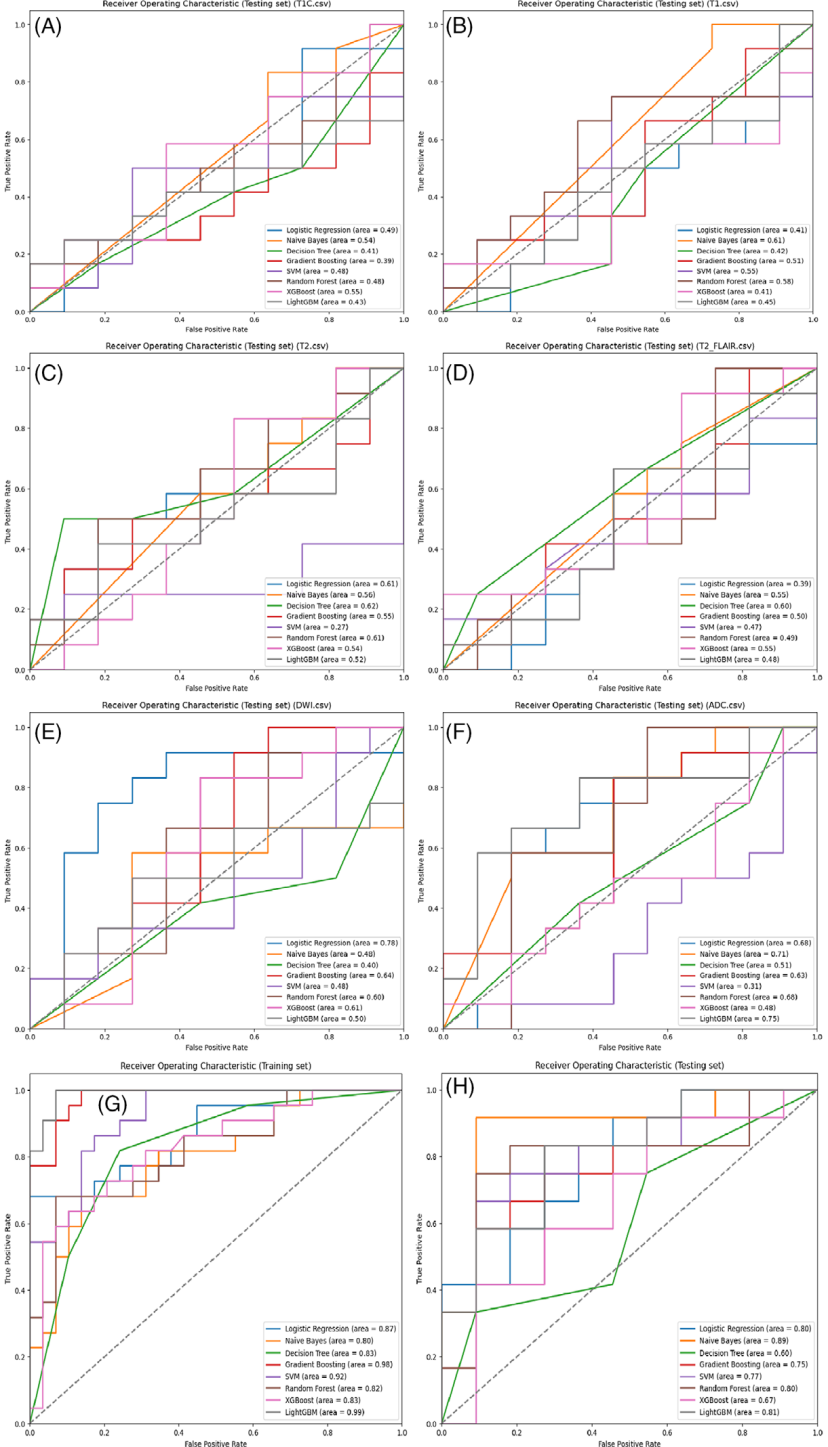
ROC curves for predicting Ki‐67 expression with different models. A‐F show ROC curves for different classifiers on single‐parameter datasets (enhanced T1, T1WI, T2WI, T2_FLAIR, DWI, and ADC) in the test set. G and H show ROC curves for various classifiers on multiparameter datasets in the training and test sets, respectively.

According to the ROC curves for the test set (Figure 3), within the single‐parameter models, the Logistic Regression classifier based on the DWI dataset and LightGBM and Naive Bayes classifiers based on the ADC dataset demonstrated better predictive capabilities, with AUCs of 0.78, 0.75, and 0.71, respectively. Other single‐parameter models generally had AUCs below 0.6. However, classifiers trained on the multiparameter dataset exhibited better discriminative abilities, with the Naive Bayes model achieving the highest AUC of 0.89 on the test set, while the Decision Tree model showed the poorest performance with an AUC of 0.6, still outperforming most single‐parameter models.

**FIGURE 4:**
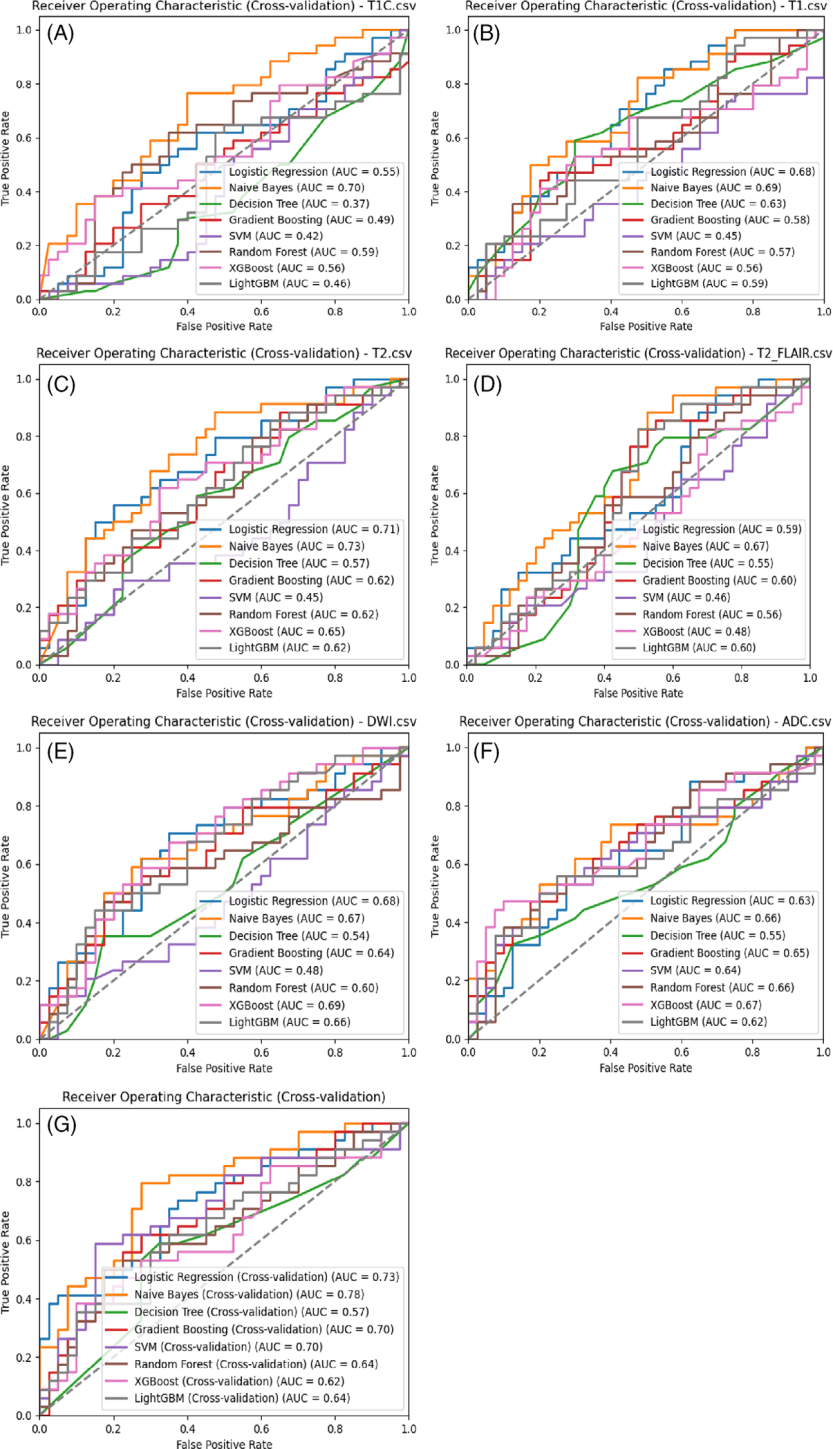
Cross‐validation ROC analysis for each model. A−F show cross‐validation ROC curves for single‐parameter models (enhanced T1, T1WI, T2WI, T2_FLAIR, DWI, ADC) with different classifiers, while G shows the cross‐validation ROC curves for multiparameter models with different classifiers.

The SVM, Random Forest, and Gradient Boosting classifiers introduced some randomness into the modeling, which led to fluctuations in the evaluation results. To obtain more robust results, 5‐fold cross‐validation was subsequently performed. As shown in Figure 4, in the single‐parameter models, the Naive Bayes and Logistic Regression classifiers based on the T2WI dataset and the Naive Bayes classifier based on the contrast‐enhanced T1 dataset demonstrated better predictive performance, with AUCs of 0.73, 0.71, and 0.70, respectively. Among the multiparameter models, the Naive Bayes, Logistic Regression, and SVM classifiers performed best, with AUCs of 0.78, 0.73, and 0.70, respectively. Overall, the AUCs of the multiparameter models were higher than those of the single‐parameter models, indicating a higher accuracy in distinguishing Ki‐67 expression levels in lymphomas.

**FIGURE 5:**
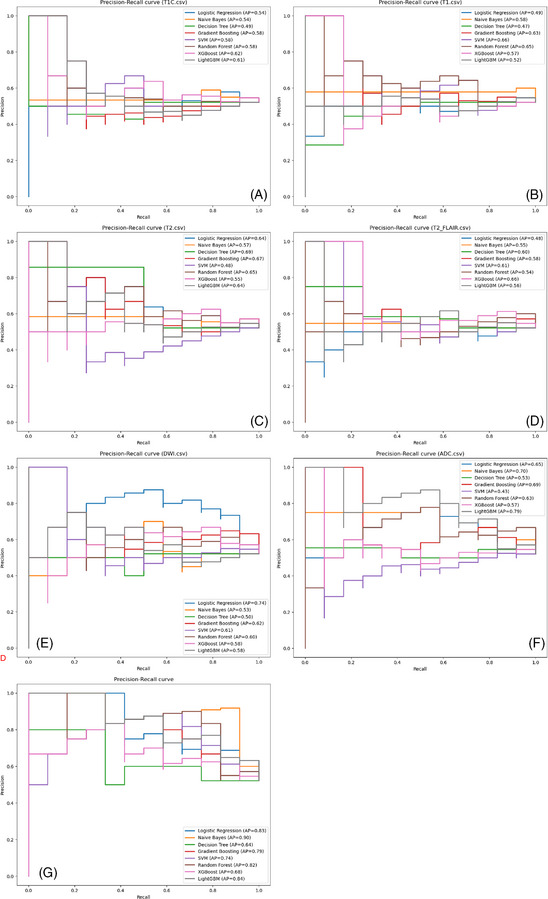
Precision–recall curve: The x‐axis represents recall (i.e., proportion of true positive results among all actual positive results), and the y‐axis represents precision (i.e., proportion of true positive results among all predicted positive results). Each point on the curve represents a precision–recall combination at a different threshold. AP denotes the area under the precision–recall curve, with values closer to 1.0 indicating better model performance.

The precision–recall curves (Figure 5) show the AP of each model's classification performance at different thresholds. In the single‐parameter models, the Logistic Regression classifier based on the DWI dataset and LightGBM and Naive Bayes classifiers based on the ADC dataset exhibited better classification performance, with APs of 0.74, 0.75, and 0.71, respectively. In the multiparameter models, the Naive Bayes, Logistic Regression, and Random Forest classifiers performed better, with APs of 0.9, 0.83, and 0.82, respectively; the Decision Tree classifier had the lowest AP at 0.64, although it still outperformed most single‐parameter models. Overall, the multiparameter models demonstrated higher AP than the single‐parameter models, indicating better classification performance.

#### 3.2.2 Intra‐group analysis of multiparameter models

Based on ROC curve analysis of the multiparameter model on the test set (Figures 3G and H), the Naive Bayes, Logistic Regression, and Random Forest classifiers demonstrated superior classification performance, with AUCs of 0.89, 0.80, and 0.80, respectively. Cross‐validation ROC curve analysis further confirmed the Naive Bayes and Logistic Regression classifiers’ performances, with AUCs of 0.78 and 0.73, respectively. In summary, the Naive Bayes and Logistic Regression classifiers performed best among the multi‐parameter models.

**FIGURE 6:**
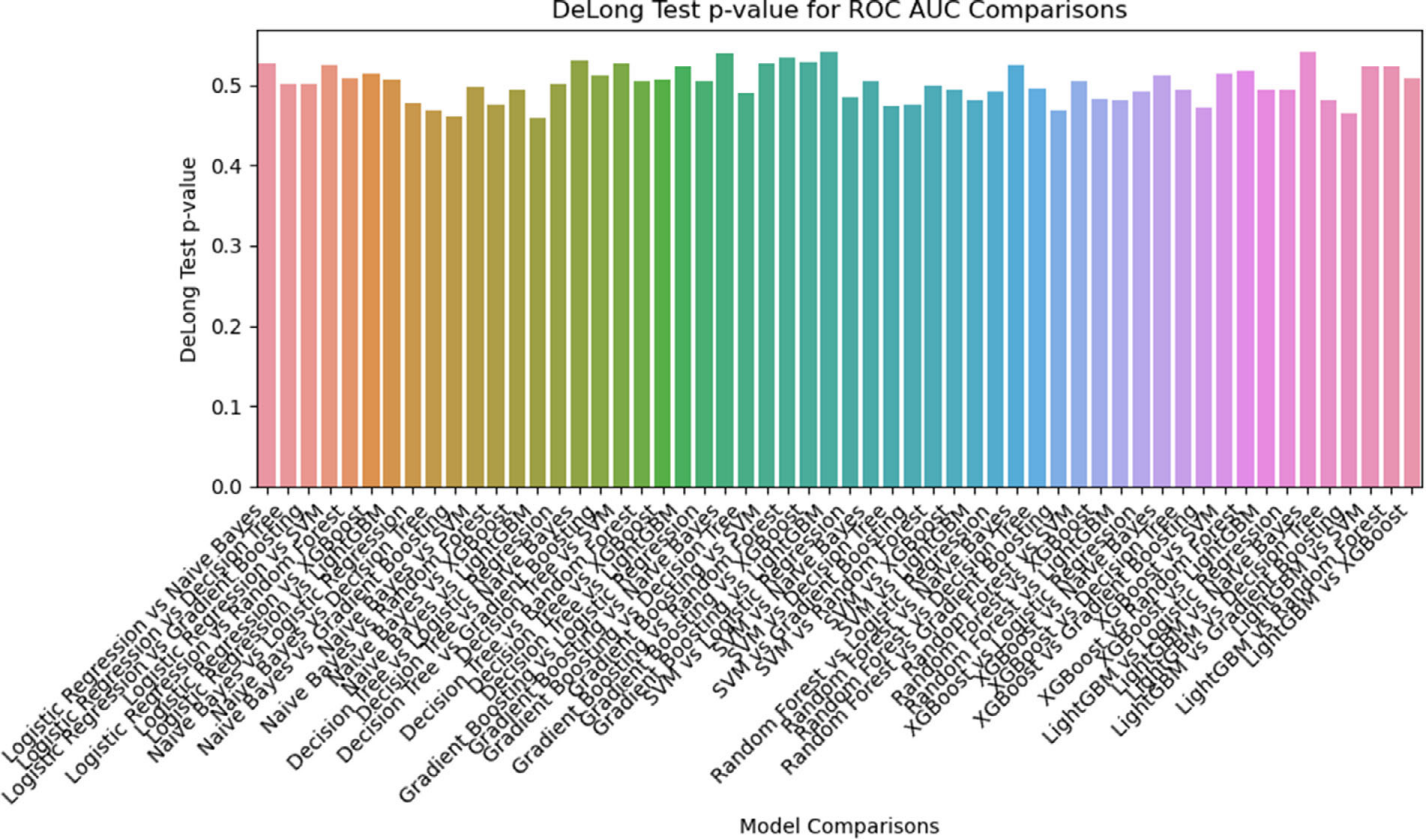
DeLong test results: The x‐axis represents comparisons between each pair of models, such as “Logistic Regression vs Naive Bayes,” indicating comparisons of their ROC AUCs. The y‐axis shows the DeLong test p‐value, reflecting the significance of the difference in AUC between models. Generally, smaller p‐values indicate more significant differences.

The DeLong test results (Figure 6) show the p‐values for the ROC‐AUC comparisons between different classifiers in the multiparameter model, determining any statistically significant difference in performance between the models. Overall, most p‐values were close to 0.5, suggesting that the differences in performance between these models may not be statistically significant.

The precision–recall curves (Figure 5G) illustrate the performance of each classifier in the multiparameter model at different thresholds. The Naive Bayes and Logistic Regression classifiers demonstrated better classification efficacy, with APs of 0.90 and 0.83, further validating their effectiveness in handling imbalanced data.

**FIGURE 7:**
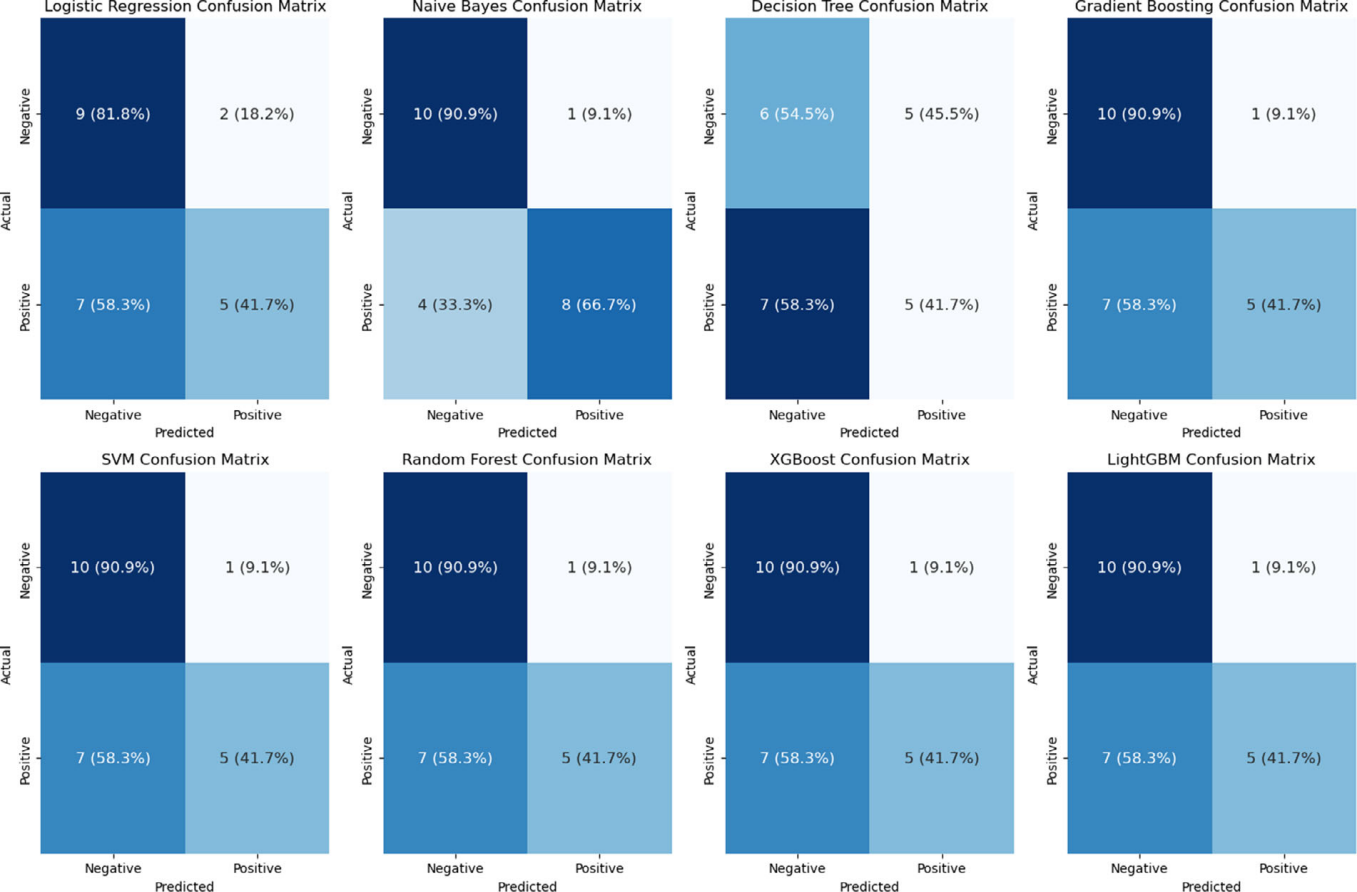
Confusion matrix of different classifiers for Ki‐67 expression level classification: The color intensity reflects the value within each cell, with darker colors indicating higher values.

The confusion matrix for the multiparameter model (Figure 7) indicates that the Naive Bayes classifier exhibited better classification performance, achieving true positive (TP) and true negative (TN) rates of 90.9% and 66.7%, respectively, whereas the other classifiers had relatively higher misclassification rates.

**FIGURE 8:**
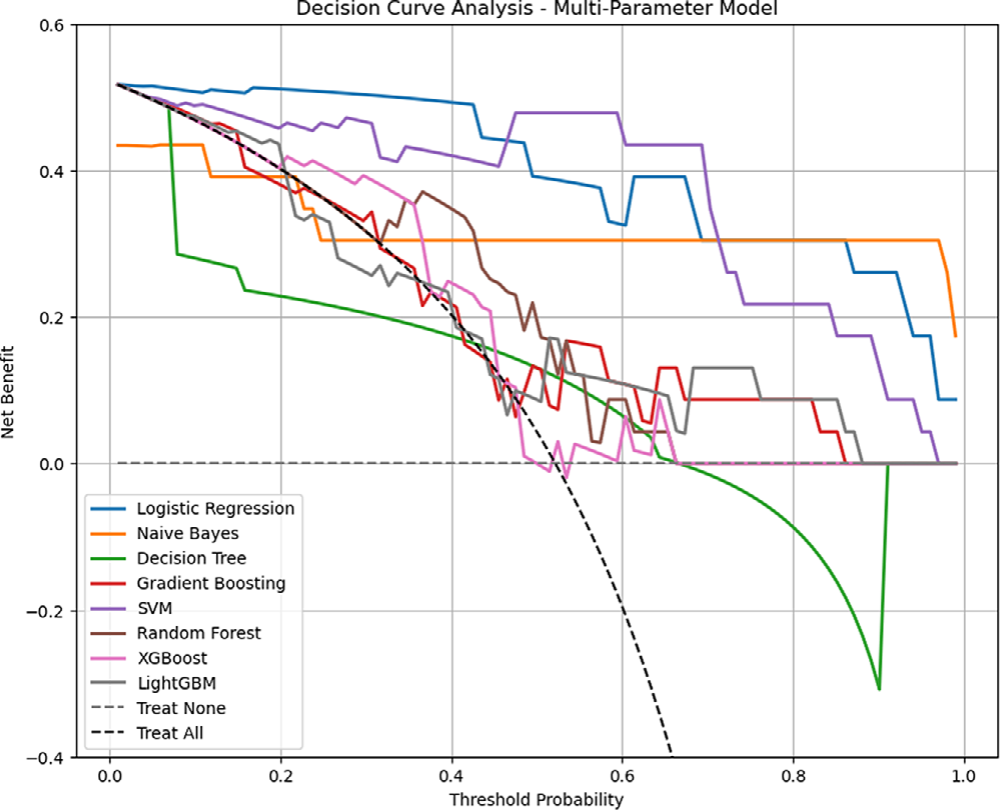
Decision curve analysis of multiple classifiers in the multiparameter model, evaluating net benefit at different thresholds: The x‐axis represents the threshold probability used for classification decisions, and the y‐axis represents the net benefit, which measures classifier utility at different thresholds by weighing correct and incorrect predictions. Each line represents a classifier, such as Logistic Regression or Naive Bayes. Lines closer to the upper‐right corner indicate better model performance. “Treat None” represents no intervention, with a net benefit of zero, shown as a flat horizontal line. A model curve above “Treat None” indicates a net benefit over no intervention. “Treat All” represents universal intervention, with net benefits decreasing as the threshold increases owing to rising false positives, resulting in a downward‐sloping line. A model curve to the right of “Treat All” suggests greater net benefit than universal intervention at that threshold.

The decision curve analysis (Figure 8) shows the net benefit of each classifier at different decision thresholds. The results indicate that most classifiers yielded similar net benefits at lower thresholds, with the differences becoming apparent as the threshold increased. Across the threshold range, the net benefit curve of the Logistic Regression classifier remained relatively high, outperforming most models, particularly in the low‐to‐medium threshold range (0–0.4). Naive Bayes maintained stable positive net benefits at thresholds above 0.3, although its overall performance was inferior to that of Logistic Regression.

By incorporating multiple performance evaluation metrics, multiparameter models demonstrated significant advantages in predicting Ki‐67 expression levels in patients with PCNSL. Among these, the Naive Bayes and Logistic Regression classifiers consistently outperformed the others across AUC, AP, confusion matrix, and decision curve analyses, highlighting their clear clinical benefits and strong potential for practical applications in clinical settings.

#### 3.2.3 Analysis of abnormal results

The LightGBM and Gradient Boosting classifiers based on the multiparameter dataset achieved AUCs of 0.99 and 0.98, respectively, on the training set, but their AUCs dropped to 0.81 and 0.75 on the test set, suggesting possible overfitting and limited generalization to new data. Possible reasons for this phenomenon are analyzed below.

In this study, a feature selection strategy based on univariate ANOVA and the SelectKBest method was determined to be the optimal approach after multiple trials. Alternative methods, such as L1 regularization, nested feature selection, and principal component analysis (PCA), were also evaluated but yielded suboptimal results. For example, L1 regularization significantly reduced the AUC of the model, likely owing to the excessive penalization of low‐weight features that were still relevant to Ki‐67 expression. Similarly, PCA resulted in the loss of important radiomic information associated with Ki‐67. In comparison, the ANOVA + SelectKBest approach retained predictive features while maintaining model performance and stability, making it the most effective choice for this study.

Despite the use of an optimized feature selection method, some models (e.g., LightGBM and Gradient Boosting) demonstrated overfitting, as evidenced by the large performance discrepancies between the training and test datasets. The following are potential reasons for this overfitting:

1. Limited Dataset Size: The dataset consisted of 74 patients from a single center, which is relatively small for training complex models. Ensemble learning methods such as LightGBM and Gradient Boosting require large datasets for effective generalization. Insufficient data may have caused these models to overfit the training set.
2. High Dimensionality of Features: Although the number of features was reduced to 10 through feature selection, the small sample size may have been insufficient to mitigate the risk of overfitting in a high‐dimensional feature space. The models may have captured noise or specific patterns unrelated to Ki‐67 expression.
3. Model Complexity: LightGBM and Gradient Boosting are highly flexible and capable of modeling complex non‐linear relationships. However, this flexibility can be a drawback when applied to small datasets, as the models may overfit to minor variations or outliers in the training data.
4. Feature Dependence: These models may have over‐relied on certain high‐weight features (e.g., texture‐related GLCM features). If the distributions of these features differ between the training and test datasets, they can result in unstable performance.

## 4 DISCUSSION

Accurate prediction of the Ki‐67 index in patients with PCNSL holds significant clinical value. The Ki‐67 expression index is associated with the clinical course and prognosis of NHL, with lower average Ki‐67 levels, typically observed in indolent lymphomas and higher levels in aggressive lymphomas. In patients with DLBCL, a 70% threshold for Ki‐67 expression can significantly differentiate patients with favorable or poor prognoses.^26^ Additionally, high Ki‐67 expression is strongly correlated with poorer survival outcomes, although this association exists primarily within specific NHL subtypes. For DLBCL patients with a high proliferation index (Ki‐67 > 70%), the IPI score remains an essential risk factor for predicting mortality. Another study demonstrated that a Ki‐67 index of ≥90% may be an independent prognostic factor for poor OS in patients with the non‐germinal center B‐cell‐like subtype.^27^

However, the optimal Ki‐67 cutoff for patients with PCNSL remains unclear, as different thresholds have been applied in studies of various PCNSL types. The model developed in this study focused on assessing Ki‐67 expression specifically in cases of PCNSL originating within the brain parenchyma. Owing to epidemiological, pathological, natural course, and treatment differences, PCNSL cases involving the spinal cord, cerebrospinal fluid, or vitreoretinal space were excluded.

Radiomics, which converts conventional CT or MR images into high‐throughput quantitative data, reflects the intrinsic pathophysiology of tumors and has been used to predict Ki‐67 indices in various tumor types. Liu et al. reported CT radiomics for predicting Ki‐67 expression levels in gastrointestinal stromal tumors, with several models achieving AUCs close to 0.9 on the test set.^28^ Another large‐scale study successfully developed radiomic features for predicting the Ki‐67 index in meningiomas, also achieving AUCs close to 0.9.^29^ Zhang et al. indicated that a dual‐sequence radiomics nomogram could aid in predicting Ki‐67 expression levels in breast cancer (AUC = 0.876).^30^ In this study, the MRI‐based multiparameter radiomic model demonstrated good performance in predicting the Ki‐67 index in PCNSL, with the Naive Bayes classifier achieving an AUC of 0.89 on the test set. Logistic Regression demonstrated the highest net benefit across most threshold probability ranges (approximately 0.2 to 0.8). Naive Bayes performed well in the low‐to‐moderate threshold ranges and exhibited stable performance across the entire threshold spectrum. The consistent and superior net benefit provided by Logistic Regression in most threshold ranges makes it the most recommended model, particularly for clinical scenarios requiring a balanced trade‐off between benefits and risks. For applications prioritizing high sensitivity, such as preoperative screening, where minimizing missed cases of high Ki‐67 expression is critical, Naive Bayes may be a better choice owing to its strong and stable performance at lower threshold ranges. Conversely, for scenarios where high specificity is crucial, such as avoiding unnecessary treatments associated with false positives, Logistic Regression offers more balanced performance and is better suited for such clinical applications. Overall, our results suggest that noninvasive radiological data can be used to predict Ki‐67 expression in PCNSL, with models integrating multiparameter features proving more effective and robust than single‐parameter models. According to the DeLong test, most p‐values between models are above 0.05, indicating that the differences in AUC are not statistically significant. However, this does not imply that the models perform identically; instead, the observed differences may not surpass the threshold of random variation. In such cases, additional evaluation metrics, such as AP and decision curve analysis, become particularly valuable. A comprehensive assessment incorporating multiple metrics, including AUC, AP, and decision curve analysis, allows for a more holistic understanding of model performance. Certain models may demonstrate advantages in these alternative metrics, such as a higher AP or greater clinical stability, even if the AUC differences are not statistically significant. Notably, if a model consistently performs well across several metrics—such as achieving high AUC, high AP, and a high net benefit—it is justifiable to select it as the “optimal model,” even without statistically significant AUC differences. Moreover, practical considerations, such as the simplicity and interpretability of a model (e.g., Logistic Regression and Naive Bayes), may make it more suitable for specific clinical applications. The primary focus of this study was to provide a noninvasive, accurate, and clinically applicable tool rather than exclusively aiming for statistical significance. Notably, some single‐parameter models based on DWI and ADC also performed well on the test set, possibly owing to the imaging techniques of diffusion sequences, which quantify water diffusivity and indirectly reflect tumor cellularity.^31^ This may correspond to the differences in cellular density associated with varying levels of Ki‐67 expression. The selected radiomic features included three original features and seven higher‐order features derived from wavelet‐transformed images and extracted based on first‐order statistics and second‐order texture features. Compared with using only the original or wavelet features, combining various radiomic feature domains provides more information and a more distinctive representation of the lesion, thereby enhancing the model classification performance.^32^ In addition, wavelet features can capture heterogeneity within the tumor.^33^ Based on the feature importance bar chart, the selected wavelet features in the radiomics model of this study mainly included the GLCM, GLSZM, and GLRLM features, which reflect structural heterogeneity and are associated with tumor heterogeneity.^34^ This could explain why these heterogeneity‐reflecting radiomic features can predict the Ki‐67 index in PCNSL.

The GLCM features quantify the spatial relationships between pixel intensities and capture the complexity of tumor texture. For instance, features such as entropy and contrast are significantly elevated in tumors with high Ki‐67 expression, reflecting increased intratumoral heterogeneity and active proliferation. These features not only reveal the complexity of the tumor microenvironment but are also closely correlated with tumor aggressiveness and treatment resistance.^35^, ^36^ Similarly, the GLSZM features analyze the size and distribution of homogeneous intensity regions within the tumor, representing regional complexity and uniformity. Studies have indicated that tumors with high Ki‐67 expression exhibit lower homogeneity and greater structural complexity, indicating that increased morphological heterogeneity is associated with higher proliferation levels.^37^ Meanwhile, the GLRLM features quantify the directionality and structural characteristics of tumor texture by analyzing the length of consecutive pixels with the same intensity. Tumors with high Ki‐67 expression typically display an increase in short‐run lengths and a decrease in long‐run lengths, suggesting greater structural density and compactness, which are closely linked to rapid proliferation and heightened heterogeneity.^38^, ^39^ Overall, the GLCM, GLSZM, and GLRLM features collectively reflect the histological heterogeneity and biological behavior of tumors from different dimensions. Their association with Ki‐67 expression not only provides quantitative insights into tumor proliferation but also supports their potential as noninvasive tools for prognostic assessment and personalized treatment decision‐making. Further integration of these radiomic features with multimodal MRI data has demonstrated improved predictive accuracy for Ki‐67 expression, offering a robust foundation for clinical evaluation and treatment planning in PCNSL.

Recently, similar studies have been published. Xiong et al. and Yang et al. developed clinical radiomics nomograms based on MRI radiomic features for the preoperative prediction of Ki‐67 expression in PCNSL. The model developed by Xiong et al. achieved an AUC of 0.877 (95% CI: 0.837–0.918) on the training set and 0.866 (95% CI: 0.774–0.957) on the external validation set.^40^ Yang et al.’s model demonstrated an AUC of 0.84 (95% CI: 0.81–0.86; p < 0.001).^41^ Both teams demonstrated strong predictive performances in their models, further validating the feasibility of this approach for clinical applications. Xiong et al. focused on feature extraction and radiomic features, confirming the utility of MRI radiomics in predicting Ki‐67 expression. Yang et al. introduced an interpretable model and nomogram that translated radiomic predictions into clinically actionable insights to enhance practical applications. By contrast, our study focused on integrating multiparameter MRI datasets (T1, T1‐CE, T2, T2‐FLAIR, DWI, and ADC) and performing a comprehensive comparison of single‐ and multiparameter models using eight machine learning classifiers. This approach highlights the differences in the predictive performance among various classifiers and datasets, providing valuable insights into their relative strengths and weaknesses. Together, these studies emphasize the potential of MRI radiomics as a noninvasive method for predicting Ki‐67 expression in PCNSL, providing a pathway for individualized treatment planning and prognostic evaluation.

## 5 LIMITATIONS AND FUTURE RESEARCH PLANS

This study had several limitations. First, as a retrospective study, it may have been subject to potential selection bias. Second, the sample size was relatively small, mainly owing to the rarity of PCNSL and the strict inclusion criteria. Third, clinical data from patients were not utilized to develop a clinical model for comprehensive evaluation and comparison, partly because the low incidence of PCNSL requires a long time to collect sufficient cases, during which medical record systems might be upgraded or changed, resulting in potential data loss. Fourth, the biological processes underlying each selected feature were not thoroughly investigated, which may require further experimental studies, such as radiogenomic analyses, to enhance our understanding of PCNSL. Fifth, an external validation set was not included, which limited the reliability assessment of the model.

Future research should focus on addressing current limitations and further advancing the clinical applicability of MRI radiomics for predicting Ki‐67 expression in PCNSL. First, expanding the sample sizes through multicenter collaborations is crucial for enhancing the robustness and generalizability of the models. This would also allow for external validation, which is essential for assessing model performance across diverse populations and imaging environments. Second, inter‐center variability in imaging protocols and scanner types should be addressed by developing harmonization and normalization techniques to standardize imaging data. Third, future studies could explore advanced feature selection methods and deep learning‐based approaches to improve predictive accuracy and model robustness. Finally, incorporating interpretable models and nomograms into clinical workflows would ensure that radiomics predictions are translated into actionable insights, thereby enhancing their usability in real‐world clinical decision‐making. Longitudinal and prospective studies are also necessary to evaluate the long‐term clinical impact of radiomics in guiding treatment and assessing prognosis.

## 6 CONCLUSION

In this study, we developed and evaluated radiomics‐based multiparameter models that integrate multi‐modal MRI data to predict Ki‐67 expression in PCNSL patients. By systematically comparing the performance of single‐ and multi‐parameter models across various machine learning classifiers, we identified Naive Bayes and Logistic Regression as the most effective models, demonstrating their superior predictive accuracy and clinical utility. These findings provide a noninvasive and precise tool for the preoperative evaluation of tumor proliferation activity, offering a valuable approach to guide personalized treatment planning and improve prognostic assessment in PCNSL.

## CONFLICT OF INTERESTS STATEMENT

All authors declare no conflicts of interest.

## ETHICS APPROVAL AND CONSENT TO PARTICIPATE

This study was conducted in accordance with the principles of the Declaration of Helsinki and approved by the Ethics Committee of Shandong Cancer Hospital and Institute (Ethics number: SDTHEC202411014). Informed consent was waived for this retrospective study.

## Supporting information



Supporting Information

Supporting Information

## Data Availability

The datasets generated or analyzed during the study are available from the corresponding author upon reasonable request. The data are not publicly available due to privacy or ethical restrictions.
